# Functional Profiling of Antibody Immune Repertoires in Convalescent Zika Virus Disease Patients

**DOI:** 10.3389/fimmu.2021.615102

**Published:** 2021-02-24

**Authors:** Ahmed S. Fahad, Morgan R. Timm, Bharat Madan, Katherine E. Burgomaster, Kimberly A. Dowd, Erica Normandin, Matías F. Gutiérrez-González, Joseph M. Pennington, Matheus Oliveira De Souza, Amy R. Henry, Farida Laboune, Lingshu Wang, David R. Ambrozak, Ingelise J. Gordon, Daniel C. Douek, Julie E. Ledgerwood, Barney S. Graham, Leda R. Castilho, Theodore C. Pierson, John R. Mascola, Brandon J. DeKosky

**Affiliations:** ^1^ Department of Pharmaceutical Chemistry, The University of Kansas, Lawrence, KS, United States; ^2^ Vaccine Research Center, National Institute of Allergy and Infectious Diseases, Bethesda, MD, United States; ^3^ Viral Pathogenesis Section, Laboratory of Viral Diseases, National Institute of Allergy and Infectious Diseases, Bethesda, MD, United States; ^4^ Federal University of Rio de Janeiro, COPPE, Cell Culture Engineering Laboratory, Rio de Janeiro, Brazil; ^5^ Department of Chemical Engineering, The University of Kansas, Lawrence, KS, United States

**Keywords:** Zika virus (ZIKV), antiviral antibodies, B cells, yeast display, next-generation sequencing

## Abstract

The re-emergence of Zika virus (ZIKV) caused widespread infections that were linked to Guillain-Barré syndrome in adults and congenital malformation in fetuses, and epidemiological data suggest that ZIKV infection can induce protective antibody responses. A more detailed understanding of anti-ZIKV antibody responses may lead to enhanced antibody discovery and improved vaccine designs against ZIKV and related flaviviruses. Here, we applied recently-invented library-scale antibody screening technologies to determine comprehensive functional molecular and genetic profiles of naturally elicited human anti-ZIKV antibodies in three convalescent individuals. We leveraged natively paired antibody yeast display and NGS to predict antibody cross-reactivities and coarse-grain antibody affinities, to perform in-depth immune profiling of IgM, IgG, and IgA antibody repertoires in peripheral blood, and to reveal virus maturation state-dependent antibody interactions. Repertoire-scale comparison of ZIKV VLP-specific and non-specific antibodies in the same individuals also showed that mean antibody somatic hypermutation levels were substantially influenced by donor-intrinsic characteristics. These data provide insights into antiviral antibody responses to ZIKV disease and outline systems-level strategies to track human antibody immune responses to emergent viral infections.

## Introduction

Zika virus (ZIKV) is predominantly a mosquito-transmitted flavivirus first isolated in Uganda in 1947 that recently re-emerged in the Americas and spread rapidly (1, 2). During the recent outbreak, ZIKV presented unique features, including a variety of congenital malformations, sexual transmission, and ability to preserve in immune-privileged sites (3, 4). In rare cases, ZIKV was associated with paralysis and polyneuropathy in infected adults, known as Guillain-Barré syndrome (5). ZIKV is closely related to other globally circulating flaviviruses, including the four dengue serotypes (DENV 1-4), West Nile virus (WNV), yellow fever virus (YFV), and Japanese encephalitis virus (JEV) (6). Zika virions contain a single-strand, positive-sense, ~11 kb RNA genome that encodes three structural proteins [capsid (C), pre-membrane (prM), and envelope (E)] and seven non-structural proteins (NS1, NS2A, NS2B, NS3, NS4A, NS4B, and NS5). The prM protein facilitates the folding and assembly of E protein shortly after synthesis and is cleaved during virus maturation (7). The E protein is subdivided into three domains (DI, DII, and DIII) and is involved in receptor binding and virus fusion (8). The ZIKV E protein is a frequent target of neutralizing antibodies which may contribute significantly to protection from infection (9–11). ZIKV virions assemble as immature virus particles on the endoplasmic reticulum and incorporate the prM and E surface proteins as heterotrimeric spikes (12). During egress from infected cells, immature particles undergo conformational changes triggered by the acidic environment of the trans Golgi network that allows cleavage of prM by the cellular protease furin. The pr peptide is released to the extracellular environment while M protein remains associated with the virion (13). E proteins of mature virions exist as antiparallel dimers arranged in a herringbone pattern (14, 15). Flavivirus infected cells release infectious particles that share structural features of both mature and immature virus particles, referred to as partially mature virions; complete maturation of the virion is not required for infectivity (14, 16).

Neutralizing antibodies can play a significant role in protection from flaviviruses (17, 18). Anti-flavivirus antibodies can be either flavivirus type-specific or cross-reactive with other flaviviruses due to conserved protein sequences across the flavivirus genus (19). Both virus-specific and cross-reactive antibodies can contribute to protection, either by direct viral neutralization or via complement and antibody effector functions (20, 21). Epitope specificity has a major influence on antibody-based protection. For example, the E-DIII epitope has been shown to be a target of potent neutralizing and protective antibodies (22–25), whereas the highly conserved E-DII fusion loop is an immunodominant epitope that can be targeted by cross-reactive antibodies with limited neutralizing capacity (8, 16). Flavivirus-reactive antibodies have the potential to enhance infection through Fc-gamma receptor interactions when bound to the virion with a stoichiometry that does not support neutralization (26–29), a phenomenon known as antibody-dependent enhancement (ADE). The recent failure of a dengue virus vaccine (CYD-TDV) to provide significant protection, and the increased risk of dengue virus disease during subsequent infection in DENV-naïve vaccinees, illustrate the challenges inherent in safe and effective flavivirus vaccine development (28, 30). Prior studies also suggest that individuals with pre-existing YFV and JEV immunity generate stronger and broader immune responses to DENV vaccination, demonstrating the potential importance of flavivirus cross-reactive immune responses in human patients (31).

Antibody discovery by FACS-based isolation of antigen-specific B cells followed by variable heavy chain (VH) and variable light chain (VL) gene cloning, sequencing, expression, and testing has catalyzed major progress in antiviral antibody discovery against flaviviruses (10, 24, 32), and for other pathogens including influenza (33, 34), Ebola (35, 36), and HIV-1 (37, 38). Recent advances in next-generation sequencing (NGS) of natively paired heavy and light chain information from large B cell repertoires (39–41), combined with profiling antibody function using display technologies, has enabled new possibilities in functional antibody repertoire analysis (42). In contrast to B-cell based FACS staining techniques like single-cell RT-PCR or single-cell transcriptomics, these native heavy:light display technologies permit the immortalization of antibody repertoires and repeated FACS screening under precise conditions to determine antibody specificities and affinities against panels of antigens (42). Thus, these high-throughput native antibody display platforms can enable a comprehensive mapping of functional antibody profiles in terms of antigen specificity, epitope specificity, and affinity (42). In this study, we performed large-scale antigen specificity and affinity profiling of natively paired anti-flavivirus antibody responses in three ZIKV convalescent patients. We mapped antibody affinity and specificity against a panel of flavivirus virus-like particles (VLPs): ZIKV wild-type [ZIKV (wt)], ZIKV immature virus-like particles [ZIKV (pr-mut)], and YFV wild-type virus-like particles [YFV (wt)]. Our data describe the genetic and functional performance of a broad range of neutralizing and non-neutralizing antibodies targeting flavivirus structural proteins as part of the immune response to ZIKV disease, and represent a comprehensive approach for profiling the molecular features of human immunity against an emergent viral infection.

## Materials and Methods

### Human Sample Collection

Peripheral blood mononuclear cells (PBMCs) were isolated from three convalescent Zika virus disease patients from Martinique in a sample collection trial conducted at the National Institutes of Health Clinical Center by the VRC, NIAID. The study was reviewed and approved by the NIAID Institutional Review Board. The U.S. Department of Health and Human Services guidelines for the protection of human research subjects were followed. All participants provided written informed consent before enrollment in NCT00067054.

### Emulsion Overlap Extension RT-PCR and Yeast Library Generation

Natively paired antibody heavy and light chain sequencing and yeast surface display library generation were performed as described previously (40, 41, 43). Briefly, B cells were isolated from cryopreserved PBMCs using Memory B Cells Isolation Kit (MACS/Miltenyi Biotec, Bergisch Gladbach, Germany) and stimulated *in vitro* using IL-2, IL-21, and co-cultured with 3T3-CD40L fibroblasts for 5 days (44). Single cells were captured in emulsion droplets, lysed, and their mRNA captured with oligo (dT)-coated magnetic beads (40, 41). Overlap-extension RT-PCR was used to link heavy and light chains and introduce two restriction sites (NheI and NcoI) between the VH and VL for yeast library generation (42). The resulting cDNA libraries were sent for Illumina sequencing of natively paired heavy and light chains as previously described (41). cDNA libraries were amplified with primers containing the yeast display vector restriction sites AscI and NotI for cloning into the yeast display vector (42). PCR products were digested with AscI and NotI and ligated into the yeast display vector. This step was performed twice with each library using either *Kappa-* or *Lambda*-specific primers and the corresponding *Kappa* or *Lambda* vector. Plasmid libraries were transformed into high-efficiency electrocompetent *E. coli*, expanded, and maxiprepped (Qiagen CompactPrep Plasmid Maxi Kit, Hilden, Germany). Plasmid libraries were subsequently digested with NheI and NcoI, purified by agarose gel extraction, and an insert containing a bidirectional Gal1/Gal10 promoter was ligated between VH and VL sequences (42) and transformed into the yeast strain AWY101 (kind gift of Eric Shusta, University of Wisconsin-Madison) using a high-efficiency homologous recombination method (42) for highly parallel native heavy and light chain yeast display.

### ZIKV VLP Production, Isolation, and Characterization

The cDNA sequence encoding the wild-type prM-E protein sequence of ZIKV “H/PF/2013” strain (Genbank #KJ776791) was codon optimized, synthetized, and cloned into plasmid VRC8400 (VRC/NIAID/NIH, Bethesda, MD, USA) at Genscript (Piscataway, NJ, USA) for expression of ZIKV (wt). In order to generate ZIKV (pr-mut) VLPs, ZIKV (wt) construct had three arginines in its wild-type furin cleavage site (ARRSRR) located between pr and M mutated to glycines (resulting in ARGSGG) in order to impair cleavage of the pr peptide, which is needed for maturation of the flavivirus particles. In both ZIKV constructs, the signal peptide used was the signal peptide from Japanese encephalitis virus (JEV). Transient transfection of HEK293F cells was carried out using FreeStyle™ 293 Expression System (Thermo Fisher Scientific, Waltham, MA, USA) according to the manufacturer’s instructions. After harvest on day 3 of transfection, cell-free supernatants were obtained by centrifugation at 5,000 *g* for 10 min followed by microfiltration using 0.45 um membranes. Prior to use in sorting experiments, ZIKV VLPs were purified by liquid chromatography in 50 mM Tris pH 8.5 buffer, using Sartobind Q membrane adsorber (Sartorius) and Captocore 700 resin (GE Healthcare), as previously described (45, 46). For confirmation of VLP size and morphology, VLPs were characterized by transmission electron microscopy (TEM) using a Hitachi 7600 microscope equipped with a lens-coupled CCD (HV = 80 kV). Prior to TEM, ZIKV VLPs purified by chromatography were further submitted to sucrose cushion ultracentrifugation (20% m/v sucrose, prepared in PBS containing 25 mM HEPES) at 175,000 *g* and 4^o^C for 4 h, and re-suspended in 100 ml of PBS.

### Yellow Fever VLP Production, Isolation, and Characterization

The cDNA sequence encoding the wild-type prM-E protein sequence of YFV “Angola71” strain (Genbank #AY968064) was codon optimized, synthetized, and cloned into plasmid VRC8400 (VRC/NIAID/NIH, Bethesda, MD, USA) at Genscript (Piscataway, NJ, USA) for expression of YFV(wt) VLPs. The signal peptide used was the wild-type signal peptide from “Angola71” YFV strain. Transient transfection of HEK293F cells, harvest and clarification were carried out as described for ZIKV VLPs. Yellow fever VLPs were purified by liquid chromatography in 50 mM Tris pH 8.5 buffer, using Sartobind Q membrane adsorber (Sartorius) and Captocore 700 resin (GE Healthcare), as previously described (45, 46).

### FACS Screening of Yeast Libraries

Yeast libraries were incubated in SGD-CAA media at 20°C for 36 h to induce Fab expression. On the day of sorting, 2 × 10^7^ yeast cells (> 10-fold coverage of library size) were washed twice with staining buffer (1× PBS with 0.5% BSA and 2 mM EDTA) and incubated with anti-FLAG FITC Clone M2 (Sigma, Missouri, USA) and either ZIKV (wt), ZIKV (pr-mut), or YFV (wt) virus-like particles for 15 min at room temperature. Then biotinylated antibody with either streptavidin-phycoerythrin (PE) or streptavidin-allophycocyanin (APC) fluorophore were added simultaneously at final concentrations of 5.3 nM and 22 nM, respectively, and incubated 15 min at room temperature with gentle agitation, to fluorescently tag VLPs. The biotinylated anti-EDIII antibody Z67 (25) and SA-APC were used to tag ZIKV (wt) VLPs, while the biotinylated anti-pr antibody 19Pr (developed at VRC/NIH) and SA-PE were used to tag ZIKV (pr-mut) VLPs. YFV (wt) VLPs were tagged with biotinylated antibody 4G2 and SA-APC. Samples were washed 3× with staining buffer, re-suspended in 0.6 ml staining buffer, and kept in the dark on ice until sorting.

A BD FACS-Aria II cell sorter running DIVA software was used to sort all FITC+/PE+ or FITC+/APC+ cells from each sample and collect them in SD-CAA media. Sorted yeast were expanded 24–48 h at 30°C and transferred to SGD-CAA media in preparation for a second round of screening. This was repeated again for a total of three rounds of screening. In Round 3, diagonal affinity gates were drawn to separate antibody populations by VLP affinity. At the first round of sorting, a sample of each yeast library was stained only with anti-FLAG FITC, and all FITC+ (i.e., VL+) cells were sorted and sequenced for use as a reference database for NGS enrichment ratio calculations. Flow cytometry data was analyzed using Flowjo9 (Flowjo, LLC, Oregon, USA).

### NGS Analysis of Sorted Yeast Libraries

Sorted yeast were expanded at 30°C for 24–48 h after each round of FACS screening. A portion of this culture was used for high-efficiency yeast plasmid DNA extraction (47). A high-fidelity polymerase (Kapa Hifi HotStart Mastermix, Kapa Biosystems, Massachusetts, USA) and primers targeting the yeast display vector backbone (**Table S4**) were used to amplify VH and VL genes from each library. A second round of primer-extension PCR with barcoded primers added a unique identifier to all amplicons from a particular library (41). Libraries were sequenced on the Illumina 2x300 platform and sequencing was repeated for each library at each round of FACS screening (Presort, VL+, Round 1, Round 2, and Round 3).

### Bioinformatic Analysis

Data processing of Illumina FASTQ files was performed as reported previously (40, 41). Briefly, Illumina sequences were quality-filtered, followed by V(D)J gene identification and CDR3 annotation using IgBLAST (48). Sequences with out-of-frame V(D)J recombination were excluded, and productive sequences were paired by Illumina read ID and compiled by exact CDR3 nucleotide match. Clustering of CDR3 nucleotide sequences was performed using vsearch v2.4.3 (49) to 96% nucleotide identity with terminal gaps ignored; VH:VL pairs with less than two reads were excluded from the final list of VH:VL clusters. Consensus sequences for all antigen-specific antibodies were generated based on exact CDR-H3 and CDR-L3 nucleotide match between paired VH:VL and separate VH and VL NGS sequencing data, as previously described (39, 43).

Antibody clonal lineages were tracked across sort rounds by their CDR-H3 amino acid sequence. The frequency (F) for a given CDR-H3 sequence (x) in a sorted sample (*y*) was calculated as:

$$F_{x,y}=\frac{{reads}_{x,y}}{sum\;of\;reads\;in\;y}$$

We also calculated the enrichment ratio (ER) of each CDR-H3 compared to the Fab-expressing (VL^+^) antibody library (Er_x,y_) for each clonal lineage in each round of sorting.

$${ER}_{x,y}=\frac{F_{x,y}}{F_{x}\;in\;{VL}^{+}\;sample}$$

CDR-H3 amino acid sequences were clustered using usearch/v5.2 (50) to identify clones with one or two amino acid differences that might be derived from sequence errors; co-clustered variants with 50-fold lower prevalence than a dominant CDR-H3 sequence in the same cluster were excluded from the final analysis.

### mAb Production and Purification

Antibody heavy and light chain sequences were appended with restriction sites for insertion into human IgG1 antibody expression vectors (42). For each antibody, Expi293F cells were co-transfected with heavy and light chain plasmids using the ExpiFectamine^TM^ 293 Transfection Kit (Thermo Fisher Scientific, Massachusetts, USA). Secreted antibody in supernatant from transient transfection were purified with Protein G or A resin (GenScript, New Jersey, USA) and concentrated using an Amicon Ultra-4 Centrifugal 30K Filter Unit (MilliporeSigma, Maryland, USA), then stored at 4°C.

### ELISA Assays

100 ng/well of ZIKV(pr-mut) VLPs were coated on ELISA plates at 4°C overnight. ELISA plates were then blocked with 300 μl of Superblock (Thermo Fisher Scientific, Waltham, MA, USA) at room temperature for 2 h. Purified antibodies were added after serially dilutions in Superblock and incubated at room temperature for 2 h. Next, 100 μl of 20,000-fold diluted IgG Fc Mouse anti-Human HRP (Invitrogen, California, USA) were added to each well and incubated for 2 h at room temperature. Plates were washed between each step with PBST (0.5% Tween-20 in PBS). Finally, 100 μl of Super AquaBlue ELISA substrate (Thermo Fisher) was added and incubated for 10 min before the reaction was stopped using 1M sulfuric acid. Absorbance was measured at 405 nm.

### Neutralization Assays

Pseudo-infectious reporter virus particle (RVP) production was performed as described previously (51). Briefly, RVPs were produced by co-transfection of a GFP-expressing WNV sub-genomic replicon with genes encoding ZIKV or YFV structural proteins (C, prM, and E) provided in trans using the Lipofectamine 3000^TM^ transfection reagent (Invitrogen, California, USA) in HEK-293T cells. The resulting standard preparation of ZIKV or YFV RVPs retain some level of uncleaved prM due to incomplete furin cleavage and contain a heterogeneous mixture of particles that display epitopes associated with both mature and immature forms of the virus. To generate more homogenously mature RVPs populations, a third plasmid expressing human furin was included in the transfection step (52). Virus-containing supernatants were harvested and filtered 3 to 7 days post-transfection. Plasmids encoding the structural genes (ZIKV, H/PF/2013; YFV, 17D-204) were generated as previously described (53). RVPs were diluted sufficiently to ensure antibody excess and incubated with an equivalent volume of serially diluted mAbs in a 96-well plate format for 1 h at 37^o^C. The resulting immune complex was then used to infect Raji cells expressing flavivirus attachment factor DC-SIGNR (Raji-DCSIGNR) and incubated at 37^o^C for 36 to 48 h. Cells were fixed with ~3% paraformaldehyde, and GFP expression was detected by flow cytometry. The resulting data were analyzed and dose-response curves were fit using nonlinear regression to calculate the antibody concentration dilution required to inhibit infection by 50% (EC_50_) (Prism 7 Software; GraphPad). To measure antibody-dependent enhancement (ADE), RVP-antibody immune complexes were generated as described above and used to infect FcγR-expressing K562 cells in a 96-well format for 36 to 48 h at 37^o^C. Cells were fixed, and infection was scored as a function of GFP expression as detected by flow cytometry. Peak amplitude ≥3-fold above background (defined as the average % GFP positive cells in the absence of mAb) was considered distinguishable enhancement; peak amplitudes below background were assigned a value half the limit of detection. At least two independent experiments were performed for neutralization and ADE assays, except for mAb-20 which was only tested once (in duplicate technical replicates) per assay due to limited material.

### Statistical Analysis

Statistical significance for repertoire-scale somatic hypermutation (SHM) and enrichment ratio data was determined using the Kolmogorov-Smirnov test on R (54) and RStudio (55), and indicated using bars and asterisks where appropriate. Non-statistically significant comparisons are not shown. SHM and ER plots in were made using ggplot2 (56). For multiple comparisons such as three different donor comparisons (Donors 1, 2, and 3) or three different pairwise isotype comparisons (IgG:IgA:IgM), a Bonferroni correction was used and a *p* value of 0.016 was considered the threshold for statistical significance.

## Results

### High-Throughput Sequencing and Yeast Display Library Generation of Natively Paired Heavy and Light Chain Antibody Repertoires

**Figure 1:**
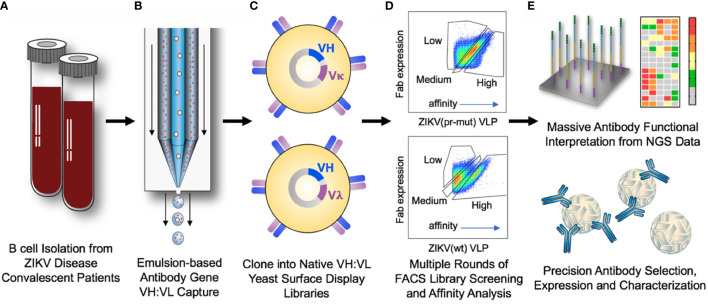
Comprehensive analysis of antibody responses to ZIKV infection. **(A)** PBMCs were collected from convalescent ZIKV disease patients between 3 and 5 weeks post-diagnosis. CD27+ antigen-experienced B cells were isolated and expanded *in vitro*. **(B)** Single antigen-experienced B cells were isolated into emulsion droplets, and overlap extension RT-PCR was used to physically link the antibody variable region heavy (VH) and light (VL) DNA sequences onto the same DNA amplicon. **(C)** Paired VH:VL antibody sequences were cloned into Igκ or Igλ display vectors and expressed as Fabs on the surface of yeast libraries for functional antibody screening. **(D)** Yeast libraries were screened by fluorescent-activated cell sorting (FACS) for binding to ZIKV (pr-mut), ZIKV (wt), and YFV (wt) virus-like particles (VLPs). In the third round of screening (shown), yeast cells were gated based on the ratio of Fab surface expression to antigen binding to bin libraries by relative affinity. **(E)** Sorted yeast populations were characterized by NGS, and library sequences were mined to determine the specificity and affinity of antibodies in the repertoire against flavivirus antigens. A subset of high-interest antibodies was selected from comprehensive antibody functional data for expression as soluble IgG and characterization of neutralization activity and ADE against ZIKV and YFV.

We obtained peripheral blood mononuclear cells (PBMCs) from three donors with confirmed ZIKV disease (**Table S1**) for profiling antibody responses against flavivirus VLPs. CD27+ antigen-experienced B cells were isolated and paired heavy and light chain variable region gene sequences (VH:VL) were captured using a high-throughput single B-cell sequencing technology, as previously described (40, 41, 43). A total of 1.45 × 10^6^ CD27+ B cells were stimulated *in vitro* and allowed to expand for emulsion-based single cell isolation and sequencing of natively paired heavy and light chains via NGS (**Table S2**). From these cell populations, 114,096 unique antibody lineages were identified after stringent quality filtering and sequence clustering, as previously described (41, 42) (**Table S3**). Natively paired heavy and light chain amplicons were cloned into surface display plasmids to generate yeast display libraries expressing antibody Fabs on their surface for functional screening via FACS (**Figure 1**) (42). Transformation efficiencies were maximized at each step of cloning into the yeast library, and 10-fold or greater coverage was achieved in all cloning steps (**Table S2**).

### FACS Screening and High-Throughput Sequencing of B Cell Repertoires

**Figure 2:**
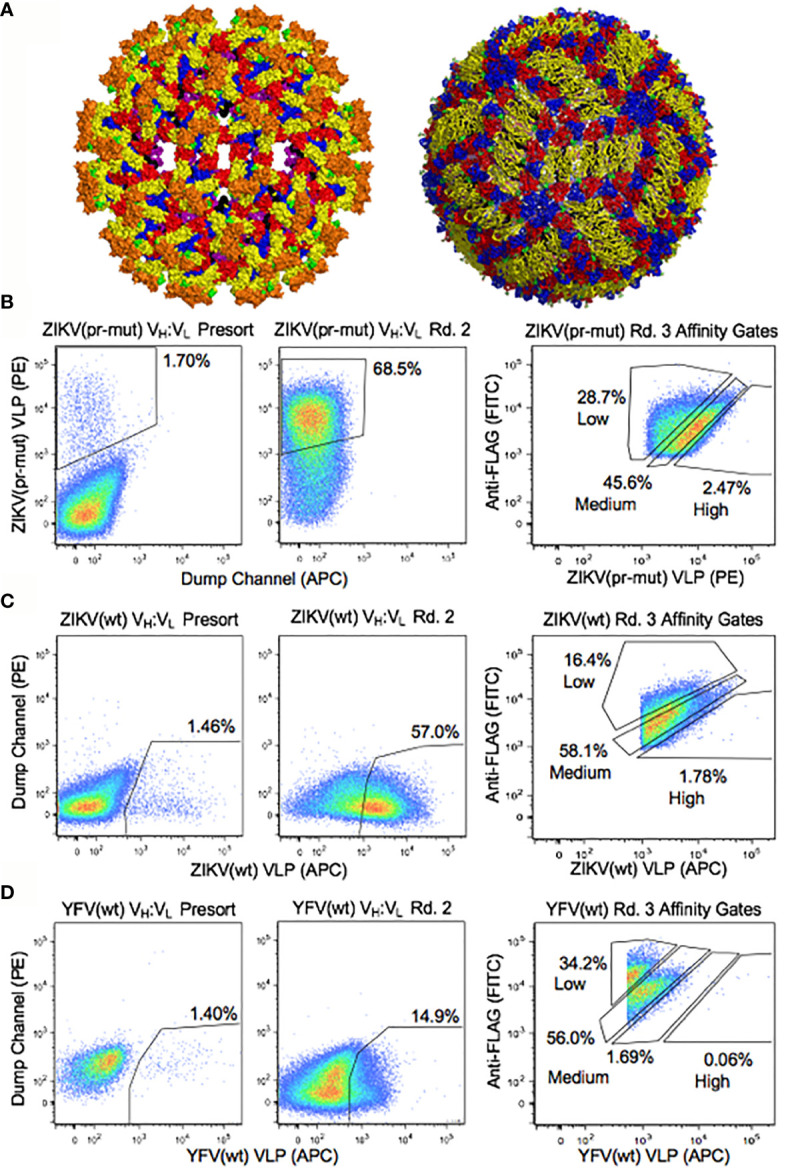
Functional analyses of yeast display repertoires for binding to flavivirus antigens. **(A)** Surface representation of ZIKV envelope proteins on the immature (left) (57) and mature (right) (58) forms of the native virus. Colors indicate E protein domain I (red), domain II (yellow) and domain III (blue), fusion loop of domain II (green), transmembrane region of E protein (purple), M protein (black), and pr domain (Orange). Functional screening was performed with virus-like particles (**Figure S1**), and the solved structures of the native virus are shown here for reference. **(B–D)** Representative FACS analysis of yeast display repertoires from B cells of ZIKV disease convalescent patients screened for binding to **(B)** ZIKV (pr-mut), **(C)** ZIKV (wt), and **(D)** YFV (wt) VLPs. FACS affinity gates were used to bin repertoires by antigen affinity.

We leveraged the renewable nature of the yeast display libraries to screen ZIKV convalescent patient B cell repertoires against three different flavivirus antigens: ZIKV (wt), ZIKV (pr-mut), and YFV (wt) VLPs (**Figure S1**). Antibody libraries were sorted by FACS after staining with flavivirus VLPs to deconvolute maturation-state specific, ZIKV-specific, and flavivirus cross-reactive antibody clones (**Figure 2** and **Figure S2**). As expected for a memory B cell response, between 1.4% and 1.7% of Fab-expressing yeast cells in the presort libraries showed binding to flavivirus VLPs (**Figures 2B–D**). After two rounds of sorting, yeast libraries were highly enriched for binding antibody variants (**Figures 2B–D**). We also performed affinity screening by fractionating yeast antibody populations by the ratio of surface Fab expression to VLP antigen binding at a single antigen concentration (**Figure 2** and **Figure S2**) (42, 59, 60). We performed NGS analysis of the yeast antibody display libraries across all stages of FACS screening to computationally determine which antibody sequences were enriched for binding to each of the three flavivirus antigens, and also to identify their respective affinities.

### NGS-Based Functional Characterization of Sorted Antibody Libraries

**Figure 3:**
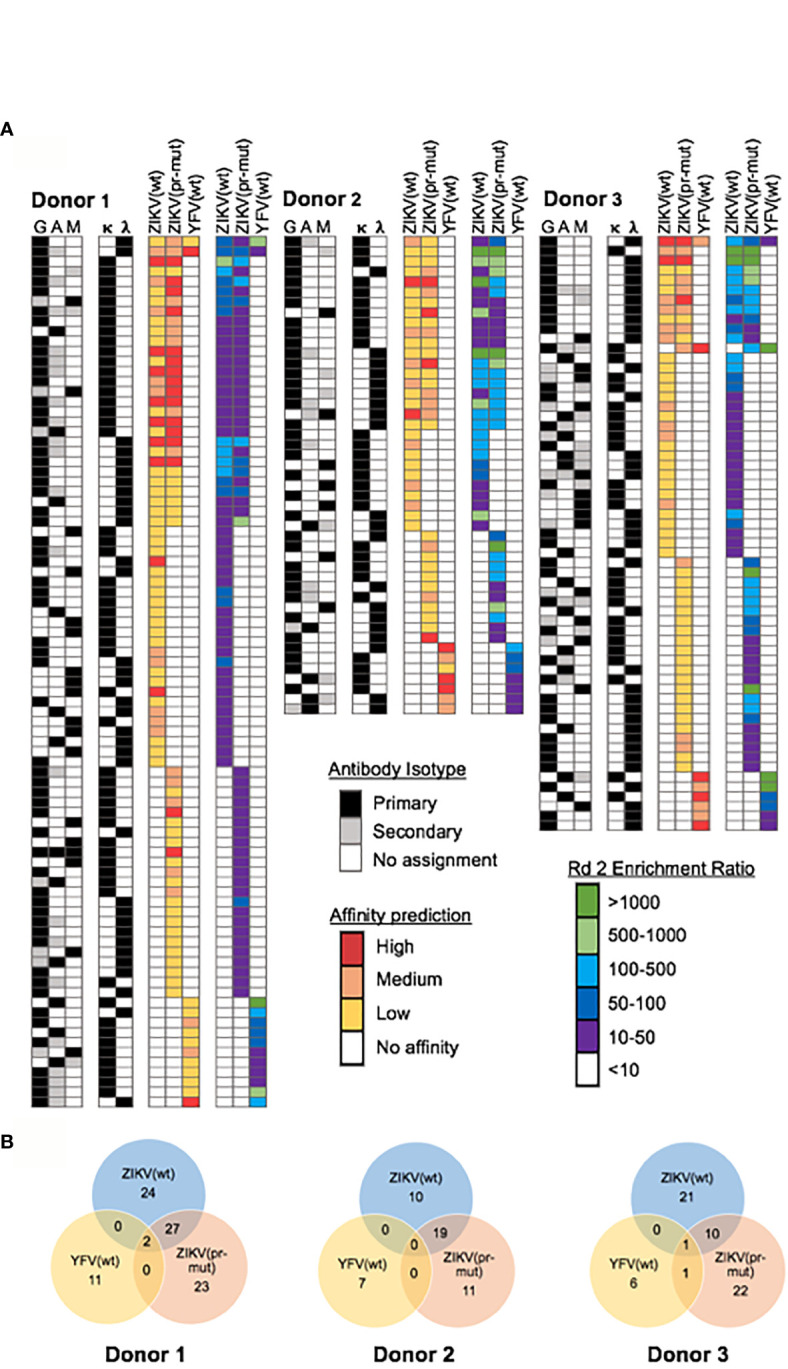
Repertoire-scale functional data for mAb lineages targeting flavivirus antigens. **(A)** Computational interrogation of yeast display NGS data after 2 rounds of sorting revealed the functional profile of anti-flavivirus antibodies in convalescent donors. Antibodies were screened against three antigens: ZIKV wild-type VLPs [ZIKV (wt)], ZIKV immature VLPs [ZIKV (pr-mut)], and YFV wild-type VLPs [YFV (wt)]. NGS data from Round 2 was used to calculate enrichment ratio and determine antigen specificity while antibody affinity was predicated based on enrichment in Round 3 affinity gates. Antibody isotype was determined from natively paired heavy:light sequence data. **(B)** Number of isolated mAb lineages and their antigen reactivity profiles for each donor.

**Figure 4:**
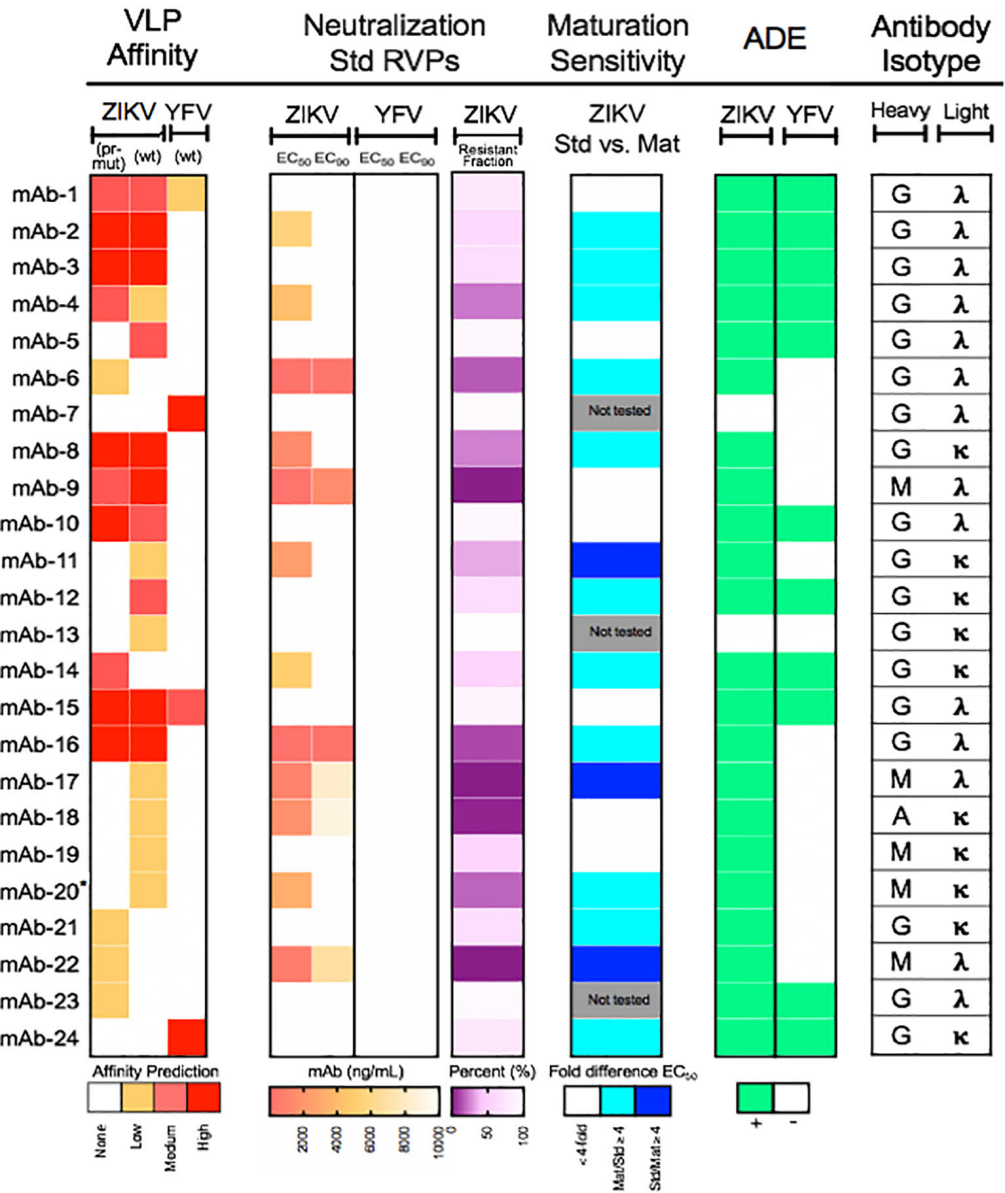
Functional characterization of a panel of monoclonal antibodies isolated from ZIKV convalescent individuals. Twenty-four mAbs isolated from ZIKV convalescent individuals were synthesized and expressed as soluble IgGs and used for flavivirus neutralization and antibody-dependent enhancement (ADE) assays. (*Left*) Heat map of computationally predicted VLP affinities against wild-type ZIKV [ZIKV (wt)], immature ZIKV [ZIKV (pr-mut)], and wild-type YFV [YFV (wt)] virus-like particles. (*Center left*) Neutralization assays were performed with the indicated mAbs using ZIKV or YFV reporter virus particles (RVPs). Data were analyzed by non-linear regression with a variable slope and constrained to the bottom to estimate the antibody concentration required to inhibit infection by 50% and 90% (EC_50_ and EC_90_, respectively). EC_50_ and EC_90_ are shown as a heat map and depicts the mean of two independent experiments performed in duplicate technical replicates. The degree of neutralization of ZIKV RVPs observed at the highest concentration of antibody tested is expressed as the percent resistant fraction. (*Center right*) The sensitivity of neutralization to the maturation state of ZIKV RVPs was analyzed for selected mAbs using standard (Std) and mature (Mat) preparations of RVPs. The EC_50_ of standard and mature RVPs was estimated for each antibody by non-linear regression analysis with a variable slope not constrained to the bottom. Maturation state sensitivity was expressed as the ratio of the EC_50_ against standard and mature ZIKV RVPs; mAbs were designated as maturation-state sensitive if the difference in titer was ≥4-fold. Data reflect the mean of two independent experiments performed in duplicate technical replicates per RVP type. Cyan is representative of mAbs that are more sensitive to partially mature virus; blue is representative of mAbs that are more sensitive to mature virus. (*Right*) Antibody binding was measured using an ADE assay performed with standard ZIKV or YFV RVPs. Antibodies capable of enhancing infection of K562 cells at three times the level of infection observed in the absence of antibody are indicated in green. Data are representative of two independent experiments performed in singlet. (*Far right*) Antibody heavy and light chain isotype for each mAb analyzed. *mAb-20 was only tested in one experiment for neutralization and ADE studies due to insufficient volume.

We performed large-scale functional mining of yeast display NGS data across all sort rounds to link functional features of antibodies (virus specificity, maturation state specificity, and coarse-grain affinity) with genetic features of the same antibody clones (including DNA sequence, V-(D-)J- gene usage, SHM, and heavy/light isotypes). First, we determined antibody specificity by tracking each antibody CDR-H3 sequence across sorted yeast libraries. Antibodies were assigned predicted specificities against ZIKV (wt), ZIKV (pr-mut), and YFV (pr-mut) separately based on enrichment after two rounds of screening, using >10-fold enrichment as a conservative cutoff value. A subset of antibody lineages bound to multiple flavivirus antigens across different screening samples (i.e., enrichment ratio >10-fold against multiple antigens), and we considered these antibody lineages to be cross-reactive against those antigens. We also predicted relative antibody affinities based on CDR-H3 sequence enrichment in the Round 3 affinity gates (either Low, Medium, and High) (42). This approach isolated 195 antigen-specific antibody lineages across the three donors with diverse flavivirus antigen specificities, predicted affinities, and isotype assignments (**Figure 3**). As expected, the majority of flavivirus-specific antibody isotypes were immunoglobulin G (IgG), and antigen-specific IgM and IgA clones were also identified. A panel of 24 flavivirus specific and cross-reactive antibodies were cloned and expressed as full-length IgG1s in HEK 293F cells. *In vitro* reporter virus particle (RVP) neutralization and ADE assays were used to assess IgG functional properties and evaluate our antigen-specific antibody predictions. 22/24 (~92%) of these antibodies were confirmed to target their predicted ZIKV and/or YFV targets by ADE assays (**Figure 4**).

**Figure 5:**
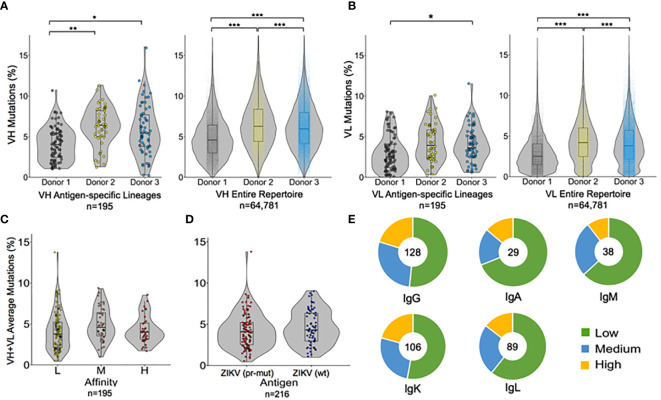
Paired genetic and functional analysis of antibody repertoire data. Percentage of somatic hypermutations in **(A)** heavy chain variable regions and **(B)** light chain variable regions, shown for each donor in both antigen-specific cells (left), and the entire repertoire (right). Each point represents a single heavy or light chain antibody lineage sequence. **(C, D)** Average SHM percentage of both VH and VL genes in the antigen-specific repertoire are reported based on **(C)** antibody affinity, or **(D)** antigen targets. **(E)** Isotype analysis for antibody lineages after separating into low-, medium-, and high-affinity groups. The total number of antibody lineages for each isotype are indicated in the center of the chart. Circles on SHM graphs represent individual points; box-and-whisker plots indicate median ± quartiles. Pairwise comparisons were performed using the K-S test and all statistically significant comparisons are noted (***p < 10^−6^, **p < 10^−3^, *p < 0.0167); any non-statistically significant comparisons are omitted.

Next, we characterized the genetic and functional features of the flavivirus-specific and non-specific antibody groups from the three human donors (**Figure 5** and **Figure S3**). Because natively paired heavy:light antibody sequencing methods capture a repertoire of antibody sequences from each patient, we had access to both flavivirus antigen-specific sequences and non-specific antibody sequences from each patient. We compared the extent of somatic hypermutation (SHM) between groups within each donor, and also across donors, and observed a similar SHM profile between VH and VL genes in the antigen-specific and non-specific repertoires across different donors (**Figure 5**). Most cross-donor SHM comparisons were statistically significant (**Figures 5A, B**), and these observations were repeated when antibody repertoires were sub-divided by isotype (**Figure S4**). Our SHM analysis suggested that the overall degree of SHM may be a feature of each individual patient, rather than being determined by antigen exposure. We did not observe statistically significant trends between SHM and affinity or antigen specificity (**Figures 5C, D**), nor was the magnitude of the enrichment ratio after Round 2 correlated closely with predicted antibody affinities (**Figure S5**). As expected, we observed some correlation between affinity and the dominant antibody isotype: IgGs encoded the largest fraction of high- and medium-affinity antibody lineages, whereas IgM and IgA clones encoded a relatively higher proportion of low-affinity antibodies (24, 61, 62) (**Figure 5E**).

### Neutralizing Antibodies Targeting ZIKV Antigens

**Figure 6:**
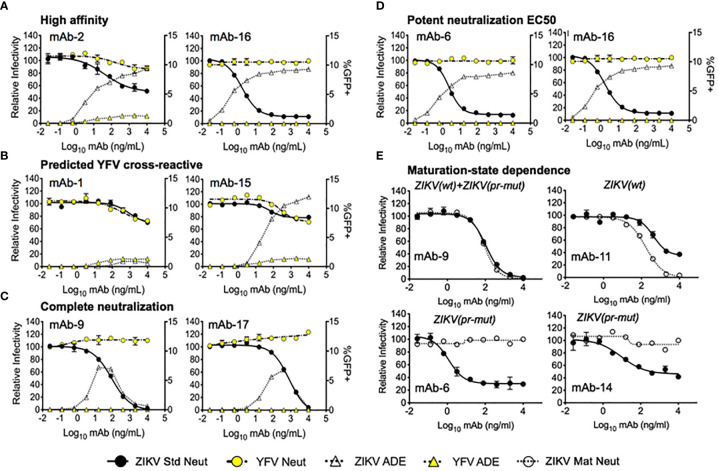
Virus neutralization and antibody-dependent enhancement of expressed mAbs. Dose-response neutralization assays were performed with the indicated mAbs against ZIKV standard (Std), mature (Mat), and YFV standard reporter virus particles (RVPs). The resulting data were analyzed by non-linear regression analysis and presented as the relative infectivity as compared to RVP infectivity observed in the absence of antibody (left y-axis). ADE was measured using standard ZIKV and YFV RVPs, and presented as the %GFP+ cells detected by flow cytometry (right y-axis). Error bars indicate the range of duplicate technical replicates. **(A)** Representative graphs of mAbs where experimental evaluation was consistent with computational predicted affinities. The highest antibody affinities shown here initiated ADE at <1 ng/ml. **(B)** Representative graphs of mAbs predicted to exhibit cross-reactive binding to both ZIKV and YFV antigens. **(C)** Complete neutralization was detected for a subset of mAbs including mAb-9 and mAb-17. **(D)** Representative curves for mAbs exhibiting single-digit ng/ml neutralization EC50. **(E)** Maturation-state dependent neutralization of a panel of selected antibodies were tested using standard and mature ZIKV RVPs. Representative graphs are shown for mAbs exhibiting no sensitivity to the virion maturation state (mAb-9), targeting mature forms of the virion (mAb-11), and targeting partially mature virus (mAb-6, mAb-14). Functional VLP targeting predictions that were determined from NGS data mining prior to neutralization assay performance are provided in italics above each neutralization curve in **(E)**.

In addition to antigen specificity, we identified several other features that were successfully predicted by our approach, as illustrated by the 24-antibody panel (**Figures 4**, **6**, and **Figure S6**). For example, mAb-2 and mAb-16 were predicted to bind with high affinity, and both showed potent ADE activity consistent with high affinity (**Figure 6A**). We further evaluated the affinity of a panel of 10 neutralizing mAbs using ELISA. We found that mAbs were generally appropriately categorized by their predicted affinities, with exception of mAb 6 (**Figure S7**). We also observed greater separation between the low affinity vs. medium+high affinity gates, which was likely a function of the single antigen staining concentration that we tested for yeast library screening (see *Discussion*).

We identified cross-reactive mAbs that neutralized both ZIKV and YFV from patients 1 and 3, who had received a prior YFV immunization (**Figure 6B**). A subset of the antibodies that we identified in the 24-antibody panel showed no resistant fraction for neutralization (**Figure 6C**) or showed potent ZIKV neutralizing EC50 (E < 2.5 ng/ml) (**Figure 6D**). Finally, we successfully predicted some maturation state-dependent antibody binding by comparing antibody binding profiles against ZIKV (wt) and ZIKV (pr-mut). To test maturation-state sensitivity, we performed neutralization assays with standard preparations of ZIKV RVPs known to retain varying levels of uncleaved prM (solid line with filled circles **Figure 6E**) and compared the results to neutralization assays using a more homogenously mature preparation of ZIKV RVPs produced in the presence of exogenous furin expression (dotted line with filled circles, **Figure 6E**). As shown in **Figure 6E**, mAb-9 was predicted to bind to both immature and mature epitopes based on our analysis of NGS screening data, and it neutralized both the standard and mature ZIKV RVP forms equally. mAb-11 was predicted to bind preferentially to mature epitopes, and *in vitro* neutralization showed that it preferentially neutralized the mature ZIKV RVP compared to the standard ZIKV RVP. Finally, mAb-6 and mAb-14 were both predicted to bind preferentially to immature epitopes; in both cases, these antibodies neutralized the standard ZIKV RVP but were unable to neutralize the fully mature ZIKV RVP. Maturation-state sensitivity assays were consistent with ZIKV affinity-based epitope predictions in 14 of the 20 tested ZIKV-targeting maturation state predictions (70%, **Figure 4**, see *Materials and Methods*.) The group of ZIKV (pr-mut)-only and ZIKV (wt)/ZIKV (pr-mut) predictions were highly consistent with maturation state assays (12/13 correct), whereas ZIKV (wt)-only predictions were less consistent with maturation state assay results (2/7 correct). These data suggest that ZIKV (wt) VLPs displayed a substantial fraction of immature epitopes (e.g., **Figure S2**), and may have also displayed cryptic immature epitopes that were not also presented by fully immature ZIKV (pr-mut). Interestingly, mAb-6 had one of the lowest ZIKV EC50 and EC90 concentrations among all antibodies that we identified, and yet it specifically targeted an immature ZIKV epitope and was unable to neutralize 100% of RVPs at the highest concentrations tested (**Figure 4** and **Figure S6**; **Table S5**). These data emphasize the role of immature surface epitopes in flavivirus neutralization dynamics and highlight the functional importance of maturation state-dependent antibody interactions.

## Discussion

Our study reports the comprehensive characterization and functional analysis of human antibody repertoires responding to ZIKV infection. This is the first investigation to combine recently developed technologies for high-throughput natively paired heavy and light chain antibody sequencing, display-based functional repertoire analysis, and computational antibody identification using NGS. Empowered by renewable yeast display libraries, we devised new computational strategies for measuring binding and estimating affinity against multiple antigens on a repertoire scale. We leveraged NGS data collection and analysis to rapidly determine functional antibody performance across three ZIKV-exposed patients, identifying new genetic and functional features of anti-ZIKV antibody responses.

We validated our antibody discovery strategies *in vitro* using a panel of antibodies with diverse functional features, showing that our approach was ~92% efficient in predicting antibody binding to flavivirus antigens. Previous studies identified panels of antibodies against flaviviruses (10, 24, 29), though our approach has several advantages compared to previously reported methods. Most importantly, the renewable nature of our natively paired heavy:light display libraries enabled us to investigate natural immune responses to ZIKV and YFV using a variety of FACS screening conditions and to isolate antibodies with different binding affinities, maturation state-dependence and cross-reactivity profiles. While antibody occupancy on the virion is not related directly to antibody affinity due to the dominant effect of unequal display of viral epitopes across and among virions, our ADE assays demonstrated the effective prediction of multiple high-affinity variants (9, 27). Our ELISA panel showed that our sorts and NGS data mining distinguished more effectively between high/medium vs. low affinity mAbs in this study (**Figure S7**), which was most likely due to our evaluation of only a single antigen staining concentration (60) which was effective for analysis of expanded clonal lineages (42) and single proteins (59) but may be less effective for diverse immune repertoire mining with a broad range of binding affinities. A broad concentration range may be particularly for anti-flavivirus antibodies with cross-reactivity across flaviviral antigens, and future studies will incorporate multiple different antigen concentrations for enhanced affinity prediction accuracy (63, 64).

We also performed the first large-scale comparison of the genetic features of antigen-specific and antigen non-specific repertoires using paired heavy and light chain genetic information. This analysis revealed that overall SHM levels appear to be substantially influenced by donor characteristics, which has major ramifications for vaccine design against other targets such as HIV, where high levels of SHM are required for effective antibody-based protection (37, 65). Future investigations will examine antigen-specific responses in spleen and bone marrow plasma cells, in addition to the antigen-experienced peripheral B cells investigated here, since our workflow does not require surface-expressed B cell receptors for antibody isolation. We will also investigate unique antibody clones from our feature-rich antibody dataset, including clonal variants within potently neutralizing lineages, as we have previously reported (42). We also observed closer correlations between antigen probe affinity and recombinant viral particle affinity in viral systems with better characterized antigens and structures, such as HIV and EBOV (42). Previous studies established that quaternary epitopes presented on the flavivirus particles could be targeted by potent neutralizing antibodies (66, 67); future studies will explore the impact of complex flaviviral multimeric and/or cryptic epitopes on mAb library screening performance. Throughout future studies we will leverage these platforms to help better understand the mechanisms and features of antibody protection conferred by infection or vaccination at a repertoire scale.

While passive prophylaxis using highly neutralizing antibodies could mitigate flavivirus disease burden, the development of an effective vaccine would be the ultimate goal for broad and low-cost ZIKV protection. We hope that in-depth analysis of anti-flavivirus antibody repertoires like we describe here may provide new insights to accelerate the development of effective antibody-based flavivirus vaccines (28). Our comprehensive approach for antibody characterization can also be leveraged for large-scale interrogation of antibody responses to other infectious and non-infectious disease targets and help us approach a more complete, systems-level functional understanding of human antibody immunity.

## Data Availability Statement

Raw sequence data is deposited in the NCBI Short Read Archive (SRA) under accession number PRJNA682833.

## Ethics Statement

The study was reviewed and approved by the NIAID Institutional Review Board. The U.S. Department of Health and Human Services guidelines for the protection of human research subjects were followed. All participants provided written informed consent before enrollment in NCT00067054.

## Author Contributions

AF, MT, KD, DD, JL, BG, LC, TP, JM, and BD, designed the experiments. AF, MT, BM, KB, KD, EN. MG-G, JP, MD, AH, FL, LW, DA, IG, and LC performed the experiments. AF, BM, KB, KD, JP, MG-G, LC, TP, and BD analyzed the data. AF, MT, and BD wrote the manuscript with feedback from all authors. All authors contributed to the article and approved the submitted version.

## Funding

This work was supported by the University of Kansas Departments of Pharmaceutical Chemistry and Chemical Engineering, by NIH grants 1DP5OD023118, 1R21AI143407, 1R21AI14440801, 1R01AI141452, P20GM113117, and P20GM103638, and by the Vaccine Research Center and the Division of Intramural Research of NIAID, NIH.

## Conflict of Interest

The authors declare that the research was conducted in the absence of any commercial or financial relationships that could be construed as a potential conflict of interest.
