# Supplementary material for: Functional Profiling of Antibody Immune Repertoires in Convalescent Zika Virus Disease Patients
Source: Front Immunol. 2021 Feb 24;12:615102. doi: 10.3389/fimmu.2021.615102 (PMC7959826; doi:10.3389/fimmu.2021.615102)
Supplement: Supplementary file 1 [file DataSheet_1.docx]

Supplementary Material

**Supplementary Figures**


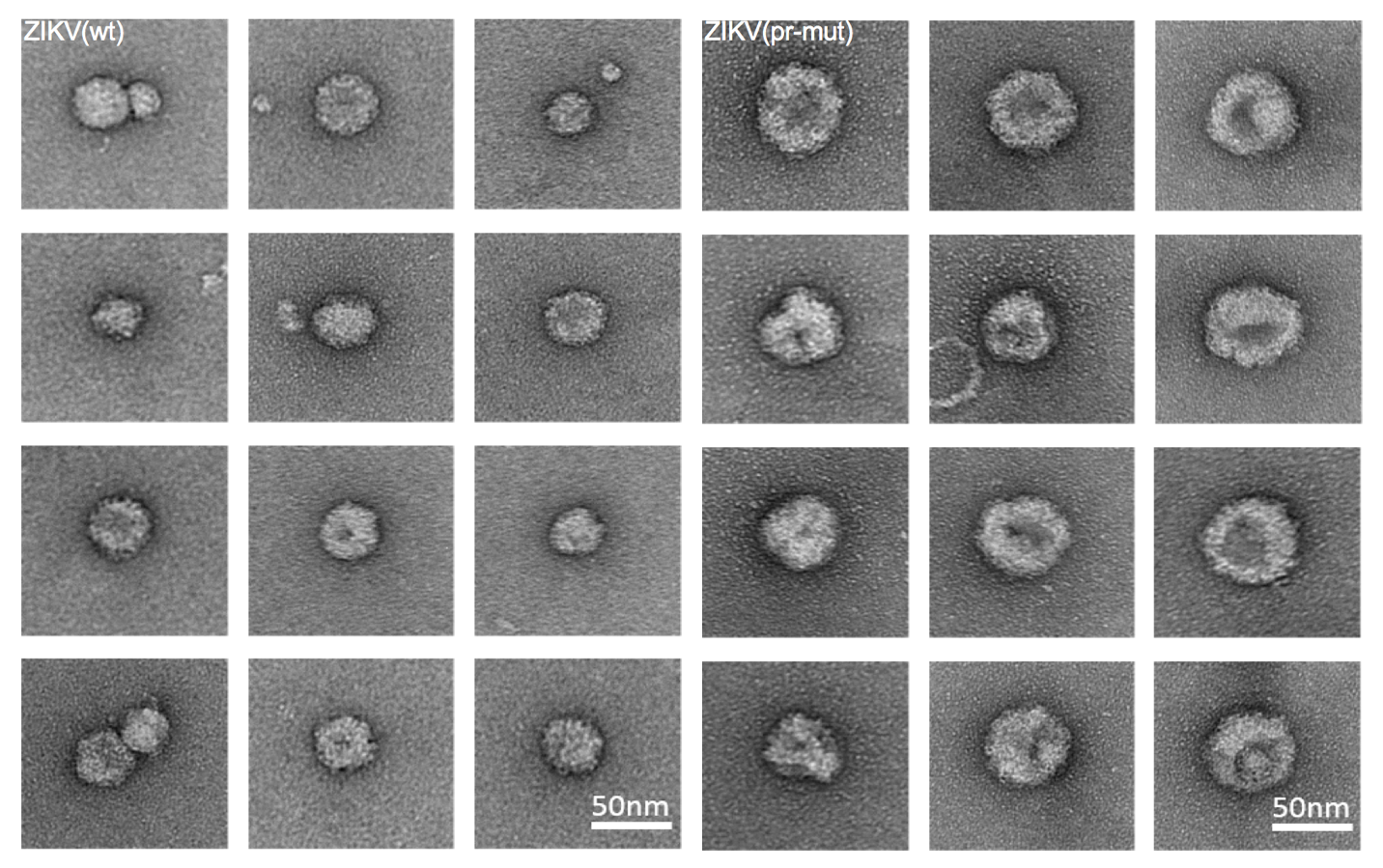


**Figure. S1.** TEM images of ZIKV VLP antigens. (A) ZIKV(wt) VLPs (wild-type), 30-50 nm diameter. (B) ZIKV (pr-mut) VLPs (furin cleavage site mutated to impair pr cleavage and particle maturation), 50-80 nm diameter. Images were taken using a Hitachi 7600 microscope equipped with a lens-coupled CCD (HV=80 kV).

**
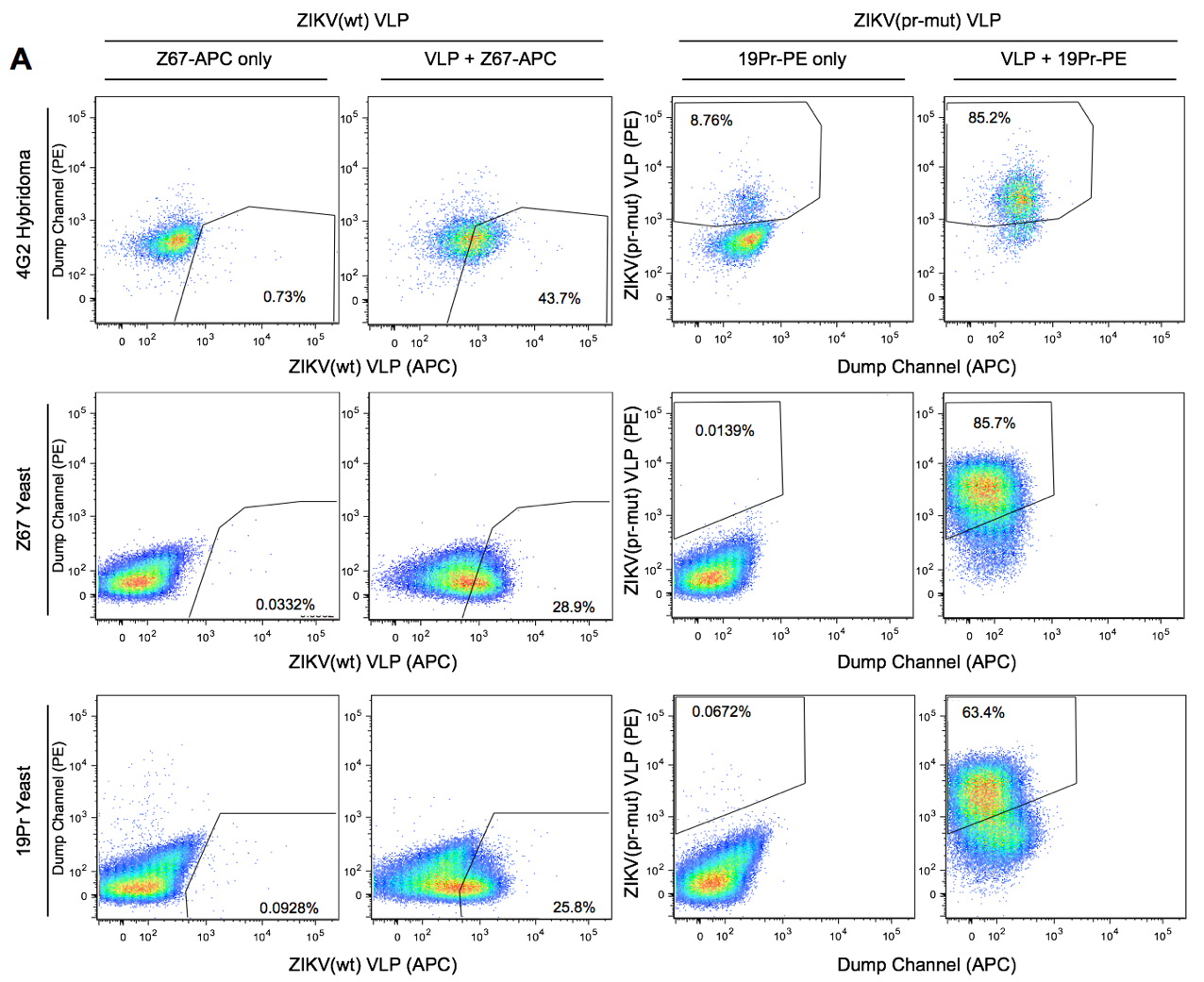
**

**
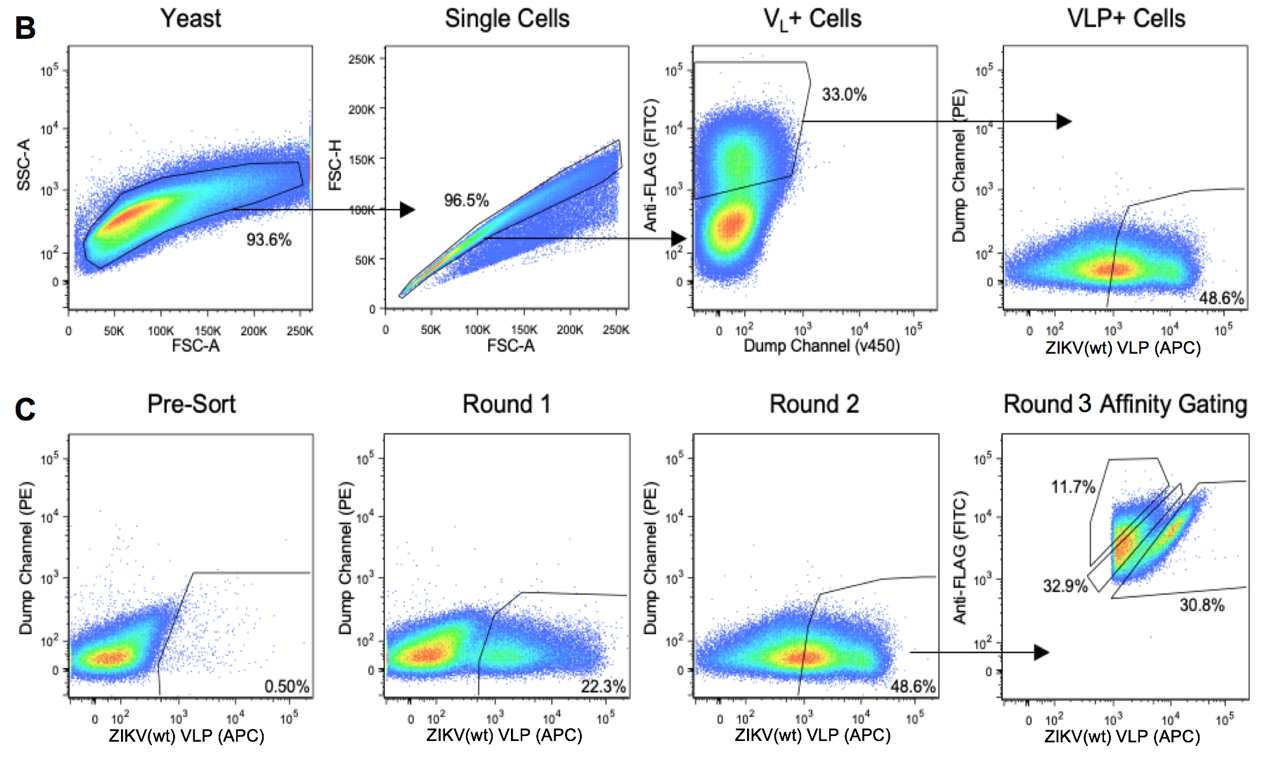
**

**Figure. S2.** **(A)** VLP probe validation with ZIKV-specific yeast and hybridoma cells. Yeast and hybridomas were stained with VLPs tagged with biotinylated antibody conjugated to streptavidin-phycoerythrin (PE) or streptavidin-allophycocyanin (APC). ZIKV (wt) VLPs were bound to mAb Z67 conjugated to SA-APC. ZIKV (pr-mut) VLPs were bound to mAb 19Pr conjugated to SA-PE. An antibody-only control was included to determine background fluorescence from mAb/fluorophore complexes. All yeast conditions were stained with 2 μg/mL anti-FLAG FITC to quantify Fab surface expression, and only FITC+ yeast are shown. **(B, C)** Representative FACS gating and sorting strategy. Yeast were stained with 2 μg/mL anti-FLAG FITC and 40 μg VLPs tagged with either SA-PE or SA-APC. (**B**) Singlet VL+ (FITC+), and VLP+ (PE or APC) yeast were bulk sorted in each round of FACS. (**C**) Sequential rounds of FACS screening enriched for VLP+ yeast. In the final screening of Round 3, diagonal gates were used to fractionate the repertoire based on relative mAb affinity.

**
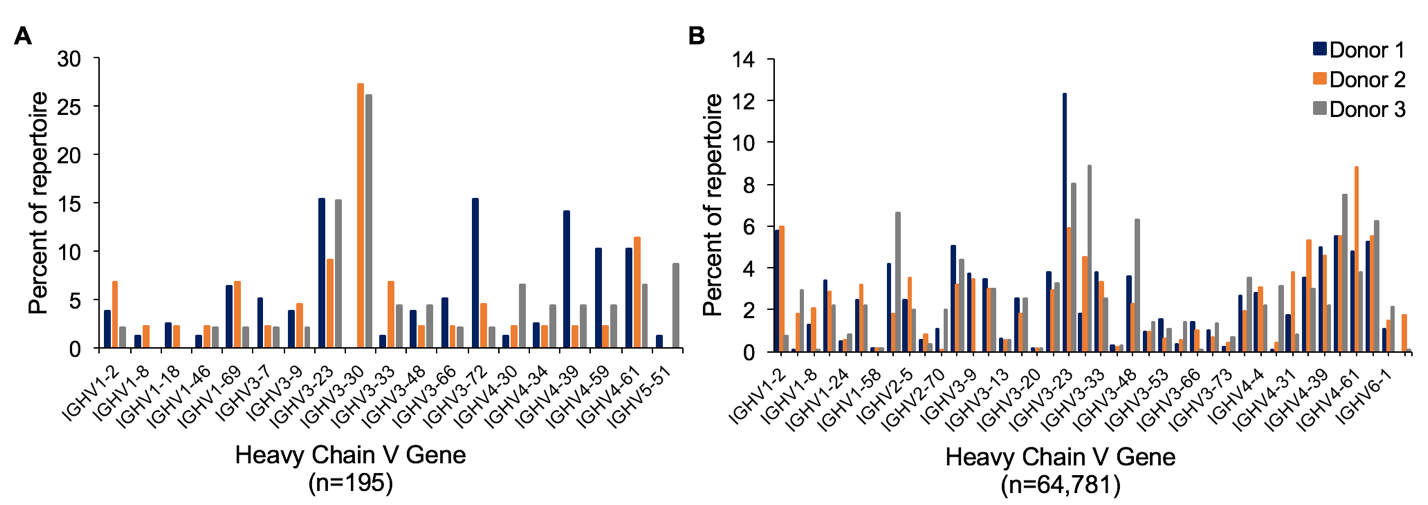

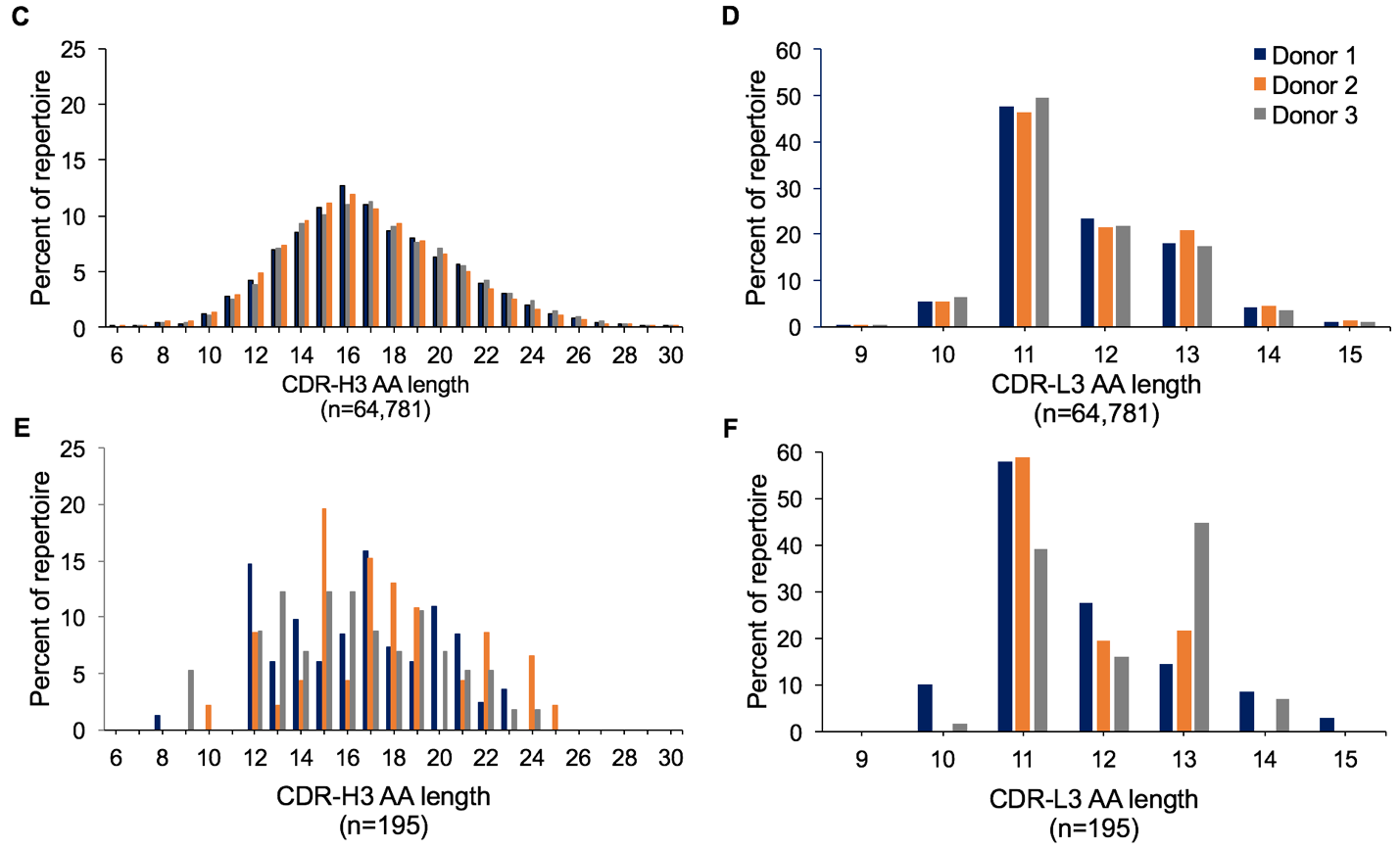
**

**Figure. S3.** VH and VL gene analyses for the three ZIKV disease convalescent donors. (**A**) VH germline V-gene distribution of flavivirus-specific B cell repertoires. (**B**) VH germline V-gene distribution for flavivirus non-specific B cell repertoires. (**C, D**) CDR-H3 and CDR-L3 amino acid (AA) length distribution for flavivirus non-specific lineages. (**E-F**) CDR-H3 and CDR-L3 AA length distribution for flavivirus-specific lineages.

**
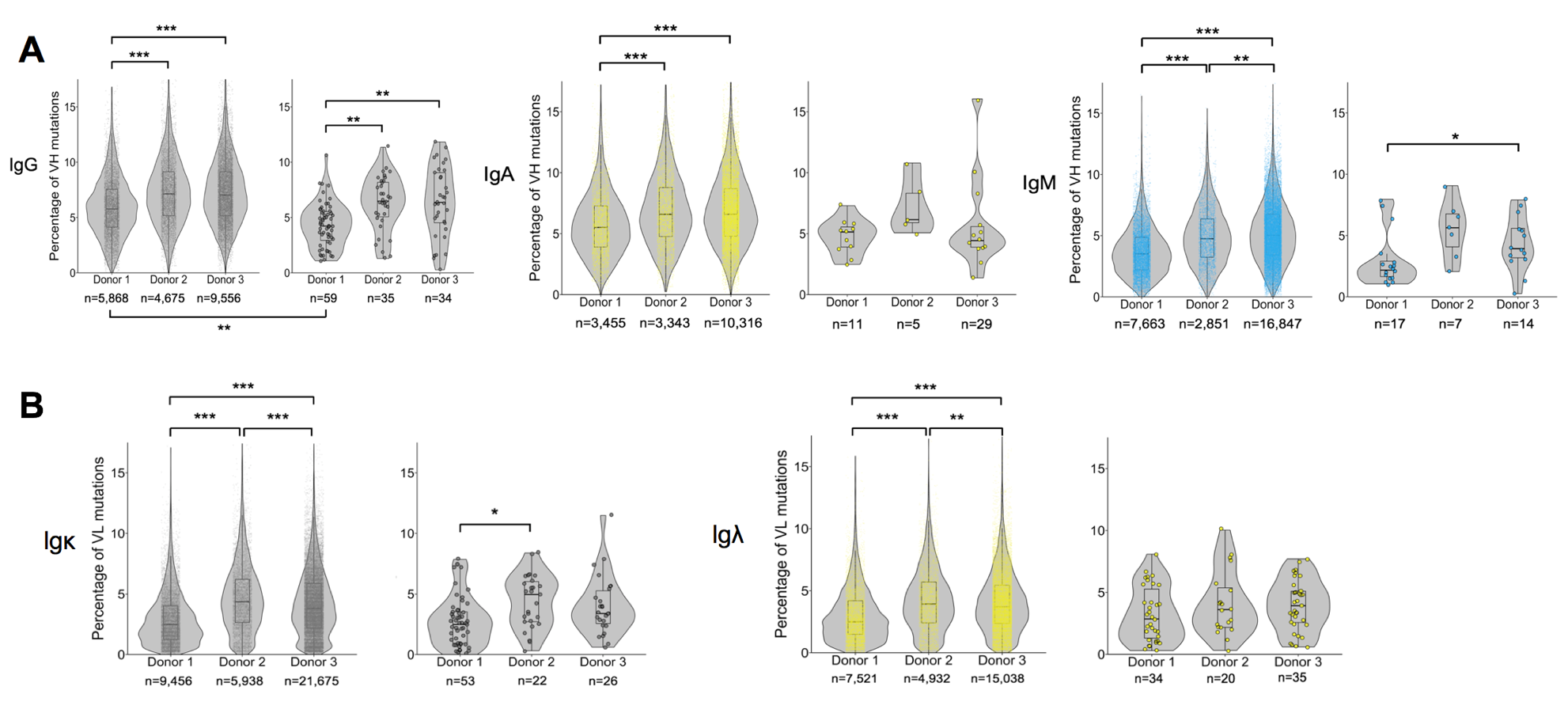
**

**Figure. S4**. Repertoire-scale somatic mutation profiles for each individual donor, for overall repertoires (left) compared to antigen-specific repertoires (right). Data are subdivided by (**A**) heavy chain isotypes, or (**B**) light chain isotypes. Each point represents a unique heavy or light chain lineage. Box-and-whisker plots indicate median ± quartiles. Pairwise comparisons were performed using the K-S test and all statistically significant comparisons are noted (****p*<10^-6^, ***p*<10^-3^, **p*<0.0167); any non-statistically significant comparisons are omitted.

**
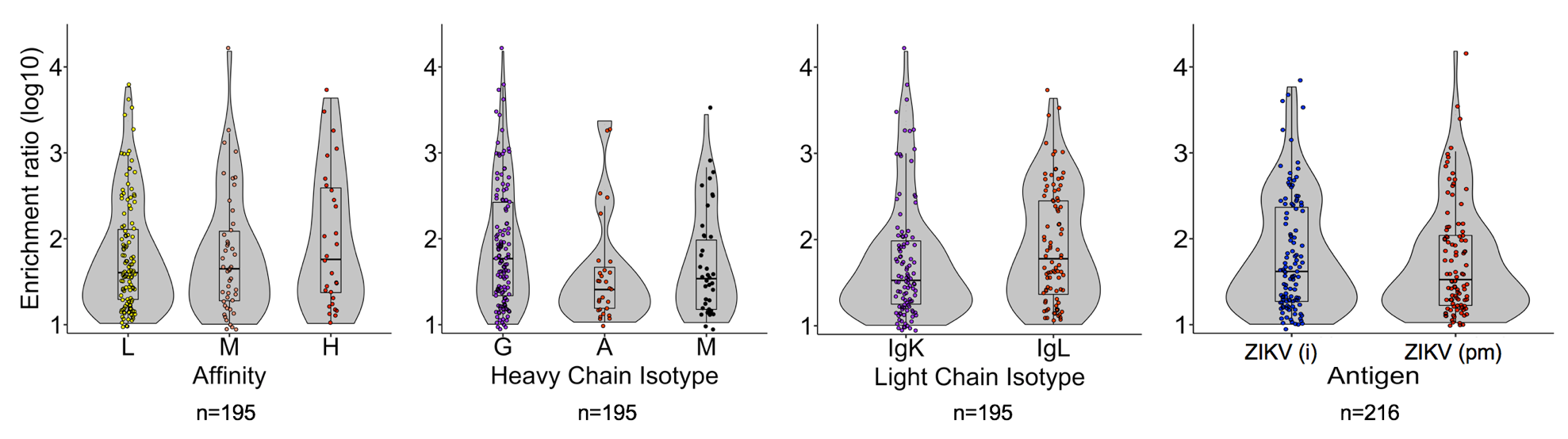
**

**Figure. S5.** Log_10_ enrichment ratios plotted according to affinity, heavy and light chain isotype, and antigen specificity for anti-flavivirus antibody lineages. Each circle represents a data point; box-and-whisker plots indicate median ± quartiles. No statistically significant pairwise comparisons were detected between groups shown here.


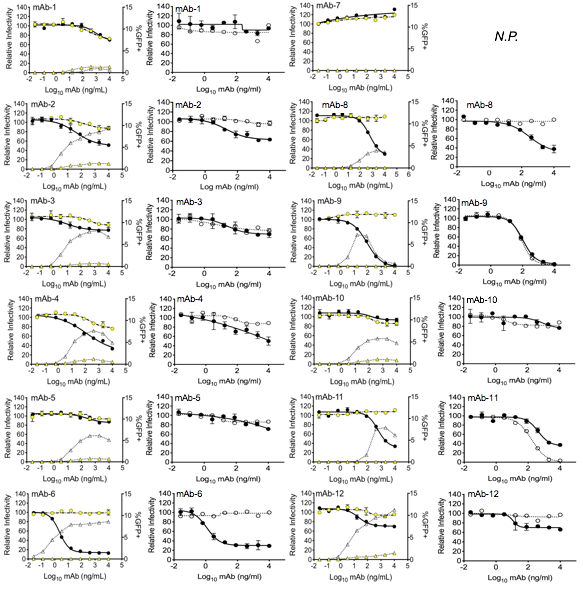


**
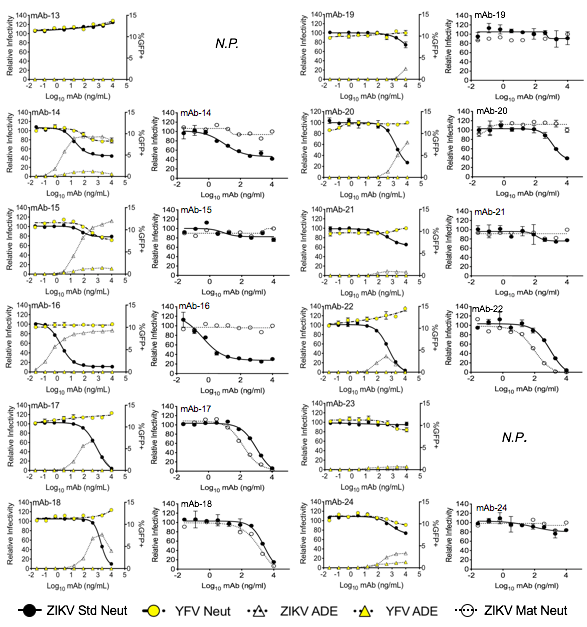
**

**Figure. S6.** **Virus neutralization and antibody-dependent enhancement of expressed mAbs.** Dose-response neutralization assays were performed with the indicated mAbs against standard (Std) ZIKV, mature (Mat) ZIKV, and standard YFV RVPs. The resulting data was analyzed by non-linear regression analysis and presented as the relative infectivity as compared to RVP infectivity observed in the absence of antibody (left y-axis). ADE was measured using standard ZIKV or YFV RVPs, and presented as the %GFP+ cells detected by flow cytometry (right y-axis). Error bars indicate the range of duplicate technical replicates. *N.P.* – maturation-state dependence assays not performed (minimal ZIKV neutralization observed).

**Figure. S7.** **ELISA characterization of anti-ZIKV mAbs.** Binding profile of 10 purified neutralizing antibodies from high affinity (H), medium affinity (M), and low affinity (L) prediction groups. Anti-ZIKV mAb Z67 was used as a positive control, and anti-HIV mAb VRC01 as a negative control.

**Supplementary Tables**

**Table S1.** Donor information for PBMC samples used in this study.

| Donor | Days post-diagnosis at blood draw | Vaccinated for YFV | Date of illness onset/symptomatic | Date of Zika diagnosis | Method of diagnosis | Sample collection date |
| --- | --- | --- | --- | --- | --- | --- |
| 1 | 21 | Yes | 01/19/16 | 01/26/16 | Urine PCR | 02/09/16 |
| 2 | 24 | No | 01/31/16 | 02/02/16 | Urine PCR | 02/24/16 |
| 3 | 37 | Yes | 02/27/16 | 03/03/16 | Urine PCR | 04/05/16 |

**Table S2.** Transformation efficiencies (TransE) for (**A**) intermediate *E. coli* vector cloning, and (**B**) yeast display library generation. 𝜅/λ ratio was approximated at 1:1.

**A**

|  |  |  |  | ***E. coli* transformation 1** | |  | ***E. coli* transformation 2** | |
| --- | --- | --- | --- | --- | --- | --- | --- | --- |
| **Library** | **Input B cells** | **Input cells emulsified per 𝜅/λ** |  | **TransE** | **Library size** |  | **TransE** | **Library size** |
| 1𝜅 | 5.5×10^5^ | 1.25×10^6^ |  | 7.65×10^7^ | 7.65×10^6^ |  | 2.01×10^8^ | 2.10×10^7^ |
| 1λ |  | 1.25×10^6^ |  | 2.58×10^8^ | 2.58×10^7^ |  | 1.55×10^8^ | 1.55×10^7^ |
| 2𝜅 | 4.0×10^5^ | 1.25×10^6^ |  | 2.63×10^7^ | 2.63×10^6^ |  | 1.40×10^7^ | 1.40×10^6^ |
| 2λ |  | 1.25×10^6^ |  | 3.87×10^7^ | 3.87×10^6^ |  | 1.40×10^7^ | 1.40×10^6^ |
| 3𝜅 | 5.0×10^5^ | 8.50×10^5^ |  | 3.32×10^7^ | 3.32×10^6^ |  | 1.28×10^8^ | 1.28×10^7^ |
| 3λ |  | 8.50×10^5^ |  | 3.49×10^7^ | 3.49×10^6^ |  | 7.00×10^7^ | 7.10×10^6^ |

**B**

|  |  |  | **Yeast transformation** | |
| --- | --- | --- | --- | --- |
| **Library** | **Input B cells** | **Input cells emulsified per 𝜅/λ** | **TransE** | **Library size** |
| 1𝜅 | 5.5×10^5^ | 1.25×10^6^ | 1.43×10^7^ | 5.70×10^7^ |
| 1λ |  | 1.25×10^6^ | 1.03×10^7^ | 4.10×10^7^ |
| 2𝜅 | 4.0×10^5^ | 1.25×10^6^ | 8.51×10^6^ | 3.40×10^7^ |
| 2λ |  | 1.25×10^6^ | 1.03×10^7^ | 4.10×10^7^ |
| 3𝜅 | 5.0×10^5^ | 8.50×10^5^ | 1.15×10^7^ | 4.60×10^7^ |
| 3λ |  | 8.50×10^5^ | 8.00×10^6^ | 3.21×10^7^ |

**Table S3.** Key statistics for next generation sequence data analysis of B cell repertoires. MiSeq data quality-filtered and clustered as previously reported (>1 exact match CDR-H3:CDR-L3 reads, 96% CDR-H3 nt clustering) (40, 41, 43).

| **Donor** | **Input B Cells** | **Total MiSeq Reads** | **Reads Passing Quality Filters** | **Unique VH:VL clusters** |
| --- | --- | --- | --- | --- |
| 1 | 5.5×10^5^ | 2,331,863 | 1,038,396 | 17,808 |
| 2 | 4.0×10^5^ | 3,748,641 | 738,512 | 33,430 |
| 3 | 5.0×10^5^ | 15,648,950 | 4,766,892 | 62,858 |

**Table S4.** Yeast library recovery primers.

| **Primer** | **Sequence** |
| --- | --- |
| 3Ydrec_huIgL_Crev_v.3.1_MSrev1 | GTCTCGTGGGCTCGGAGATGTGTATAAGAGACAG NNNN TGTAACGCTGGGGGCGGCCGC |
| 2Ydrec_huIgK_Crev_v2.4_MSrev1 | GTCTCGTGGGCTCGGAGATGTGTATAAGAGACAG NNNN ATGGCGGGAAGATGAAGACAGA |
| 2Ydrec_light_Vfor_MSfor1 | TCGTCGGCAGCGTCAGATGTGTATAAGAGACAG NNNN GAGAGGCTGCATCCGCCAT |
| 2Ydrec_heavy_Vfor_MSrev1 | TCTCGTGGGCTCGGAGATGTGTATAAGAGACAG NNNN CTGTTATTGCTAGCGTTTTAGCA |
| 2YDrec_huIgH_Crev_MSfor1 | TCGTCGGCAGCGTCAGATGTGTATAAGAGACAG NNNN AAGGCGCGCCTGTACTTGC |

**Table S5. EC50 and EC90 neutralization values.** ZIKV standard RVPs were assayed twice in Figure S6: once in the left column with ADE and YFV neutralization (ZIKV Std #1), and a second time for maturation-state dependence assays in the right column (ZIKV Std #2).

|  | **ZIKV Std #1** | | **YFV** | | **ZIKV Std #2** | | **ZIKV Mature** | |
| --- | --- | --- | --- | --- | --- | --- | --- | --- |
|  | *EC50* | *EC90* | *EC50* | *EC90* | *EC50* | *EC90* | *EC50* | *EC90* |
| mAb-1 | - | - | - | - | - | - | - | - |
| mAb-2 | 5,540 | - | - | - | - | - | - | - |
| mAb-3 | - | - | - | - | - | - | - | - |
| mAb-4 | 4,180 | - | - | - | 6,040 | - | - | - |
| mAb-5 | - | - | - | - | - | - | - | - |
| mAb-6 | 1.5 | 238 | - | - | 14.3 | - | - | - |
| mAb-7 | - | - | - | - | *N.P.* | *N.P.* | *N.P.* | *N.P.* |
| mAb-8 | 1,280 | - | - | - | 2,400 | - | - | - |
| mAb-9 | 105 | 1,390 | - | - | 94.7 | 937 | 66.0 | 404 |
| mAb-10 | - | - | - | - | - | - | - | - |
| mAb-11 | 2,430 | - | - | - | 2,820 | - | 144 | 1920 |
| mAb-12 | - | - | - | - | - | - | - | - |
| mAb-13 | - | - | - | - | *N.P.* | *N.P.* | *N.P.* | *N.P.* |
| mAb-14 | 5,060 | - | - | - | 5,530 | - | - | - |
| mAb-15 | - | - | - | - | - | - | - | - |
| mAb-16 | 1.5 | 78.5 | - | - | 23.5 | - | - | - |
| mAb-17 | 847 | 8,170 | - | - | 899 | 8,980 | 230. | 1,980 |
| mAb-18 | 1,650 | 8,880 | - | - | 2,230 | - | 1,020 | 9,290 |
| mAb-19 | - | - | - | - | - | - | - | - |
| mAb-20 | 3,280 | - | - | - | 4,660 | - | - | - |
| mAb-21 | - | - | - | - | - | - | - | - |
| mAb-22 | 625 | 6,760 | - | - | 816 | 7,820 | 79.7 | 866 |
| mAb-23 | - | - | - | - | *N.P.* | *N.P.* | *N.P.* | *N.P.* |
| mAb-24 | - | - | - | - | - | - | - | - |

*-, did not neutralize to indicated value*

*N.P., maturation-state dependence assays not performed*
